# Osteomyelitis of the Clivus Secondary to Mucormycosis

**DOI:** 10.7759/cureus.41367

**Published:** 2023-07-04

**Authors:** Ramiz S Ahmad, Davin J Evanson, Michael A Romeo

**Affiliations:** 1 Department of Radiology, Drexel University College of Medicine, Wyomissing, USA; 2 Division of Diagnostic Radiology, Reading Hospital, West Reading, USA

**Keywords:** ct (computed tomography) imaging, skull base, clivus, mucormycosis, osteomyelitis

## Abstract

Osteomyelitis of the clivus secondary to mucormycosis is a rare infection of the clivus bone due to infiltration by fungi of the genus *Mucor. *Immunocompromised patients and/or those with diabetes mellitus are most at risk of developing this disease. Here, we present the case of a 63-year-old male patient with findings of gas within the clivus on computed tomography angiography. Diagnosis of mucormycosis osteomyelitis was confirmed after endoscopic biopsy and histopathologic examination. Furthermore, as this condition is very difficult to detect on imaging, we emphasize the discussion of typical radiology findings associated with this disease based on this and other case reports in the literature.

## Introduction

Mucormycosis is a rare and severe fungal infection caused by a group of molds known as mucormycetes within the order Mucorales. Immunocompromised and/or metabolically impaired patients tend to be most at risk of fungal infection. A past medical history of diabetes mellitus is commonly seen in individuals with such infections. However, mucormycosis has also been reported in patients without a significant past medical history [1]. Importantly, SARS-CoV-2 infection may also be a predisposing factor [2]. Mucormycosis can be angioinvasive and lead to tissue infarction, necrosis, and thrombosis. Invasive mucormycosis has a relatively high mortality rate that increases with disease progression. Although various body systems can be impacted, rhino-orbital-cerebral mucormycosis (ROCM) is the most common clinical presentation of this disease [1].

Skull bones can be affected by mucormycosis after the infection has penetrated the sinonasal, orbital, and deep facial soft tissues. Clival osteomyelitis is an infection of the clivus bone located in the skull base. This pathology is considered to be a type of skull base osteomyelitis (SBO) [3]. The rare occurrence of clival osteomyelitis has been caused by *Pseudomonas aeruginosa* or *Aspergillus*; however, mucormycosis has been noted in a number of cases with rhinocerebral complications [4].

Diagnosis of osteomyelitis of the clivus secondary to mucormycosis is typically multifaceted, including findings in the patient history, physical examination, laboratory results, pathogen identification, and radiological evidence [3]. Given the rare incidence, high mortality rate, and substantial difficulty in diagnosing mucormycosis osteomyelitis, the following patient case and discussion will primarily focus on the integral role of imaging in detecting this lethal condition.

## Case presentation

A 63-year-old male patient with a history of hyperglycemia presented with weakness and a fall. Upon physical examination, a right-sided facial droop was observed. Of note, the patient had recently tested positive for SARS-CoV-2 in the emergency department. Initial non-contrast CT scan demonstrated advanced sinusitis. Upon admission to the hospital, the patient was diagnosed with diabetes mellitus. Computed tomography angiography (CTA) of the head and neck was performed as part of the diagnostic workup.

**Figure 1:**
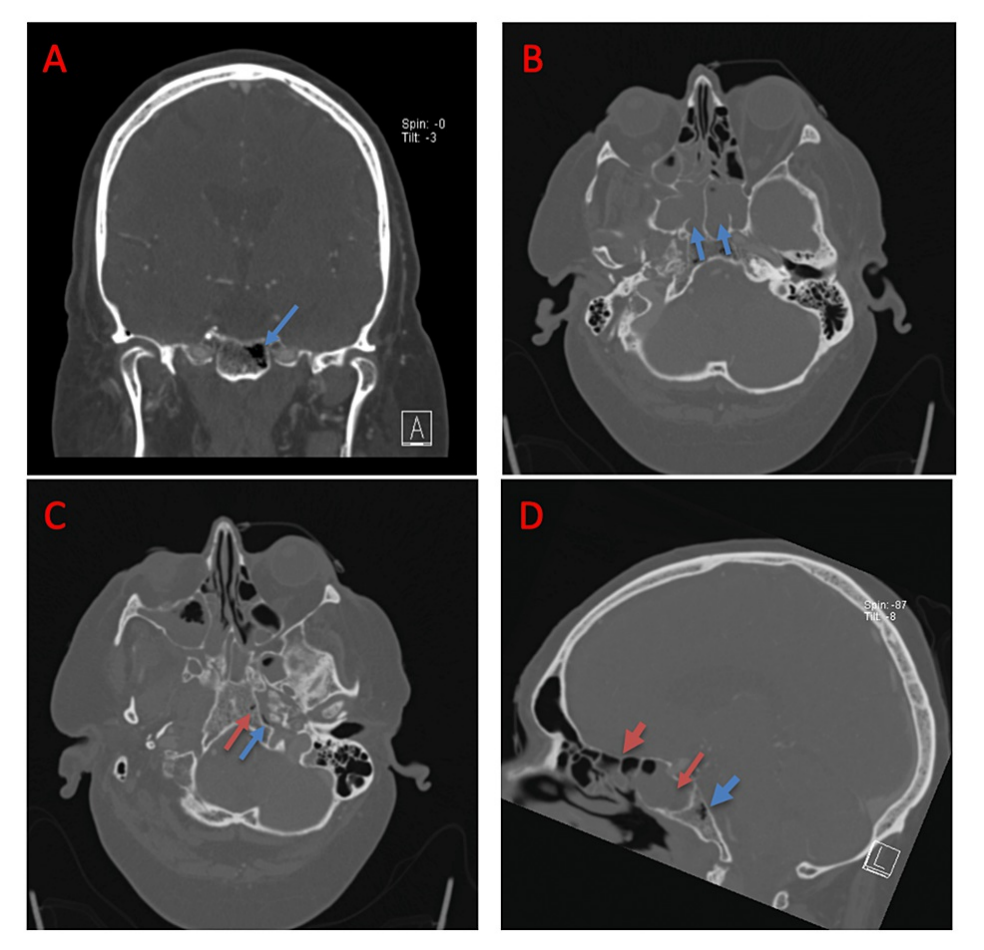
Computed tomography angiography of the head and neck. Head computed tomography angiography demonstrates the presence of gas within the clivus on the coronal section (A). The axial section demonstrates sinusitis and particularly pronounced sphenoid sinus disease (B). The axial section reveals the presence of gas within the clivus (red arrow) and the presence of curvilinear gas in the soft tissues anterolateral to the clivus (blue arrow) (C). The sagittal section (D) reveals patchy sinus opacification (red arrows) and redemonstrates gas within the clivus (blue arrow).

CTA findings are shown in Figure 1. Advanced sinusitis is demonstrated and progressed since the initial non-contrast CT was completed. Abnormal gas was seen in several foci in the skull base, left occipital condyle, and soft tissues adjacent to the clivus. Gas in these regions is highly unusual and must raise concern for osteomyelitis, possibly from a fungal source such as mucormycosis. The most appropriate clinical setting for this differential is in a patient with diabetes mellitus and significant sinusitis, as in this case.

Given the radiological findings of potential *Mucor*, a fiberoptic nasal endoscopy was performed, which demonstrated sinusitis that, in conjunction with the radiological findings of SBO, increased the suspicion of invasive sinusitis due to mucormycosis. Pathological findings reported non-septate branching hyphae, indicative of potential mucormycosis from fungal infiltration. The patient was initially started on amphotericin B but was switched to posaconazole due to concern for pancytopenia. However, repeat CT imaging revealed worsening sinusitis. As the patient began to clinically deteriorate, he was restarted on amphotericin B 6 mg/kg every 24 hours, with the dosage eventually increased to 8 mg/kg. Additionally, the patient was transferred to undergo endoscopic debridement, which demonstrated *Mucor* on the carotid wall. Unfortunately, due to the high mortality rate of osteomyelitis, the patient decompensated and died about a week later, three weeks following the initial CTA finding of mucormycosis.

## Discussion

Osteomyelitis of the clivus secondary to mucormycosis, as seen in this patient, is a rare diagnosis reported in the literature potentially due to the anatomic location of this pathology. Imaging modalities used in diagnosing mucormycosis are more commonly discussed in the context of ROCM and pulmonary mucormycosis.

Case reports in the literature regarding SBO or ROCM have mostly utilized CT and/or MRI as imaging methods. CT scans are the preferred imaging modality for detecting erosion of the sinus bones due to *Mucor*. However, regarding the invasion of soft tissues in the sinuses and beyond, MRI has been demonstrated to be more sensitive. It is possible to not detect these pathologies via imaging if mucormycosis progression is early enough [5]. Moreover, initial CT may be inconclusive, thereby emphasizing the importance of subsequent MRI or repeated CT in the proper clinical setting [6]. MRI can demonstrate abnormal bone marrow signaling of the clivus, raising concern for osteomyelitis [4]. The addition of gadolinium with MRI has been shown to further elucidate irregular enhancements of bone marrow within the skull base [7]. In some cases, CT demonstrates additional findings such as the thickening of extraocular muscles or subtle sinus mucosal thickening [4,5]. Further, CT can also display a characteristic “moth-eaten” appearance of the clivus, indicating partial destruction of the skull base [8].

CTA in patients affected by SBO mucormycosis has been used to diagnose thrombosis in head and neck vessels [9], which is a significant complication of mucormycosis, but this method can also be used to delineate the extent of clivus damage due to fungal invasion [3]. As visualized in this patient, CTA revealed the presence of intramedullary gas within the clivus, which is highly suspicious for osteomyelitis. Gas is typically much better visualized on CT rather than MRI [10].

The findings in our patient were atypical for osteomyelitis of the clivus as the bone was intact, whereas it is usually destroyed in other cases. Rather, foci of gas were visualized in abnormal locations, and this presence of gas continued to worsen without evidence of clivus bone erosion on follow-up CT imaging. MRI was not performed in this case because the intramedullary gas visualized on CT, in the presence of severe sinusitis, warranted urgent consultation with otolaryngology for biopsy and subsequent pathology evaluation. Given our patient’s initial presentation of weakness and right-sided facial droop, CTA was performed to rule out stroke. However, in cases where SBO is discovered on non-contrast CT or MRI, CTA may be indicated to diagnose thrombosis in the head and neck vasculature. In our patient, there was no evidence of thrombosis in these blood vessels.

## Conclusions

In the context of an immunocompromised or metabolically impaired patient, mucormycosis osteomyelitis is a diagnosis of exclusion when intramedullary gas is shown within the clivus on a CT. If the initial CT scan is inconclusive, then a follow-up MRI may be appropriate for higher sensitivity evaluation and reduced radiation exposure. Due to the rapid progression of this disease, further workup and treatment such as surgical debridement and administration of antifungal medication should be done urgently to improve clinical outcomes.
